# Supplementary material for: Estimating Economic Losses Caused by COVID-19 under Multiple Control Measure Scenarios with a Coupled Infectious Disease—Economic Model: A Case Study in Wuhan, China
Source: Int J Environ Res Public Health. 2021 Nov 9;18(22):11753. doi: 10.3390/ijerph182211753 (PMC8621982; doi:10.3390/ijerph182211753)
Supplement: Supplementary file 1 [file ijerph-18-11753-s001.zip › ijerph-1406284-supplementary.pdf]

International Journal of
Environmental Research and Public Health

MDPI

Article

Estimating Economic Losses Caused by COVID-19 under Multiple Control Measure Scenarios with a Coupled Infectious Disease—Economic Model: A Case Study in Wuhan, China

Xingtian Chen [1,2], Wei Gong [1,2,*], Xiaoxu Wu [3,*] and Wenwu Zhao [1,2]

[1] State Key Laboratory of Earth Surface Processes and Resource Ecology, Faculty of Geographical Science, Beijing Normal University, Beijing, 100875, Beijing, China; 201921051033@mail.bnu.edu.cn (X.C.); zhaoww@bnu.edu.cn (W.Z.)
[2] Institute of Land Surface System and Sustainable Development, Faculty of Geographical Science, Beijing Normal University, Beijing, 100875, Beijing, China
[3] State Key Laboratory of Remote Sensing Science, College of Global Change and Earth System Science, Beijing Normal University, Beijing, 100875, Beijing, China
* Correspondence: gongwei2012@bnu.edu.cn (W.G.); wuxx@bnu.edu.cn (X.W.)

Citation: Chen, X.; Gong, W.; Wu, X.; Zhao, W. Estimating Economic Losses Caused by COVID-19 under Multiple Control Measure Scenarios with a Coupled Infectious Disease – Economic Model: A case study in Wuhan, China. *Int. J. Environ. Res. Public Health* **2021**, *18*, x. https://doi.org/10.3390/xxxxx

Academic Editors: Subhash Pokhrel; Doug Coyle

Received: 18 September 2021
Accepted: 01 November 2021
Published: date

Publisher's Note: MDPI stays neutral with regard to jurisdictional claims in published maps and institutional affiliations.

Copyright: © 2021 by the authors. Submitted for possible open access publication under the terms and conditions of the Creative Commons Attribution (CC BY) license (http://creativecommons.org/licenses/by/4.0/).

Supplementary Materials

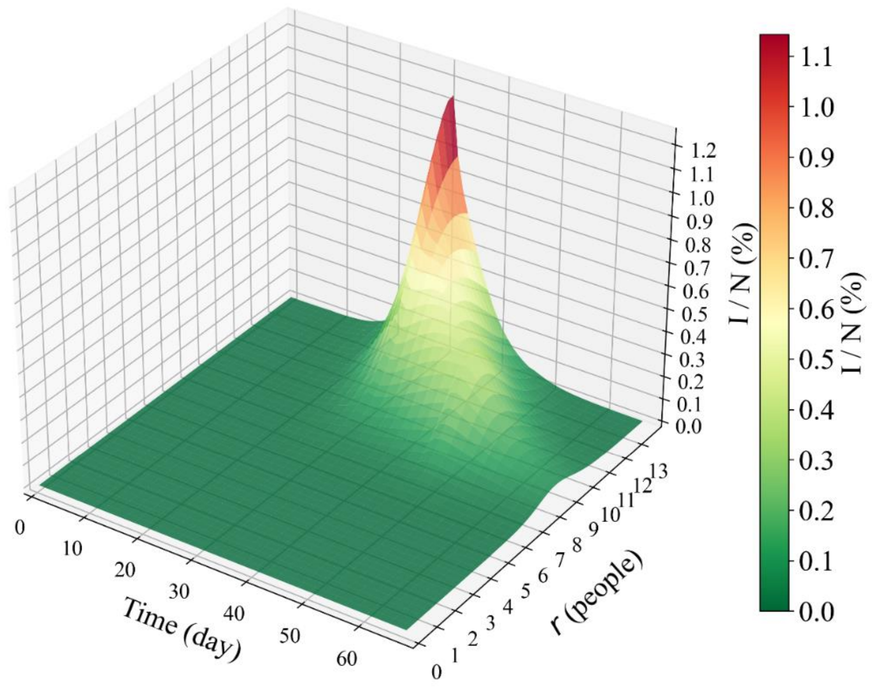

Figure S1. The five-stage model simulates infected patients in different scenarios of the effective numbers of daily contacts (r), where I is the number of infected patients; N is the total population.

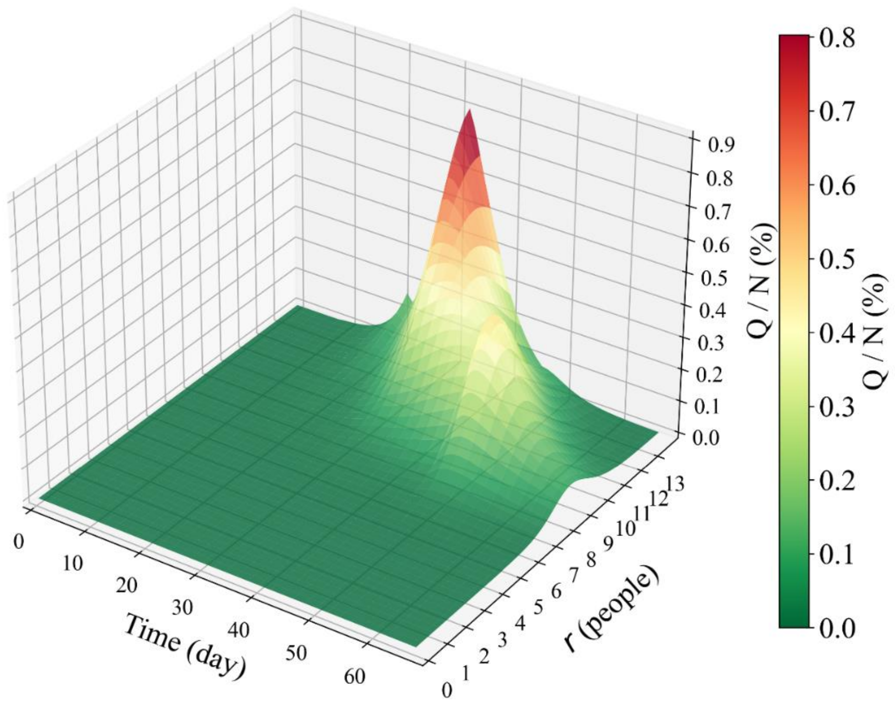

Figure S2. The five-stage model simulates quarantined patients in different scenarios of the effective numbers of daily contacts (r), where Q is the number of quarantined patients; N is the total population.

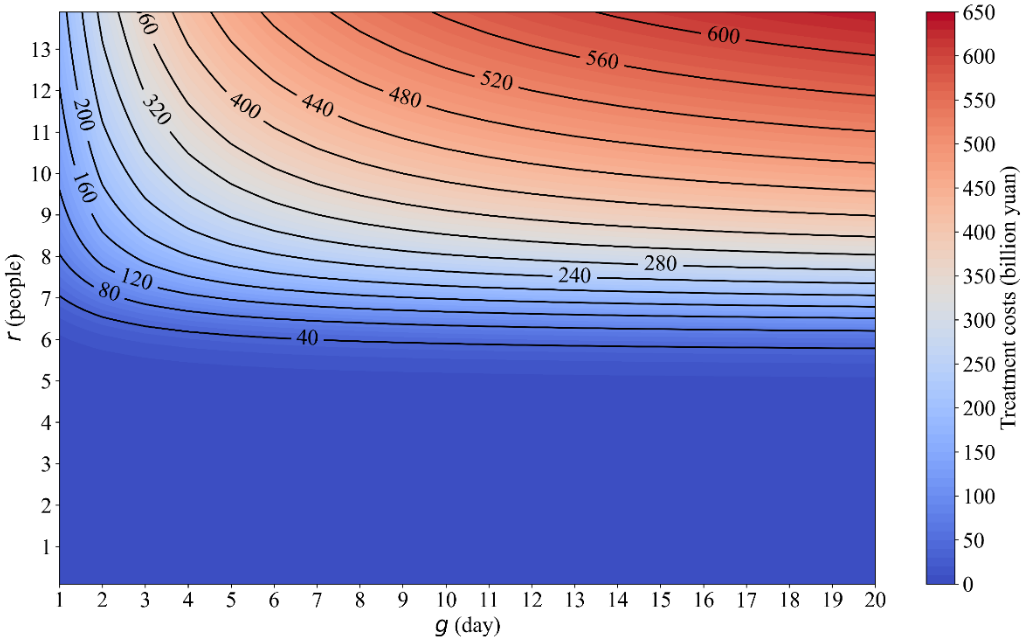

Figure S3. Treatment costs under various control measures, where r is the effective number of daily contacts; g is the average waiting time for quarantined patients.

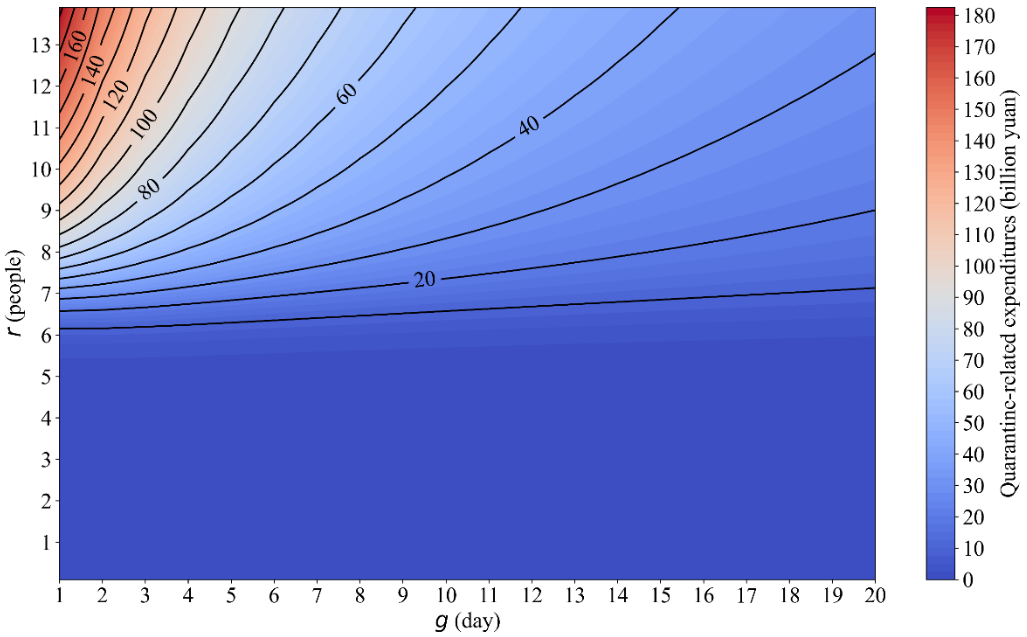

Figure S4. Quarantine-related expenditures under various control measures, where r is the effective number of daily contacts; g is the average waiting time for quarantined patients.

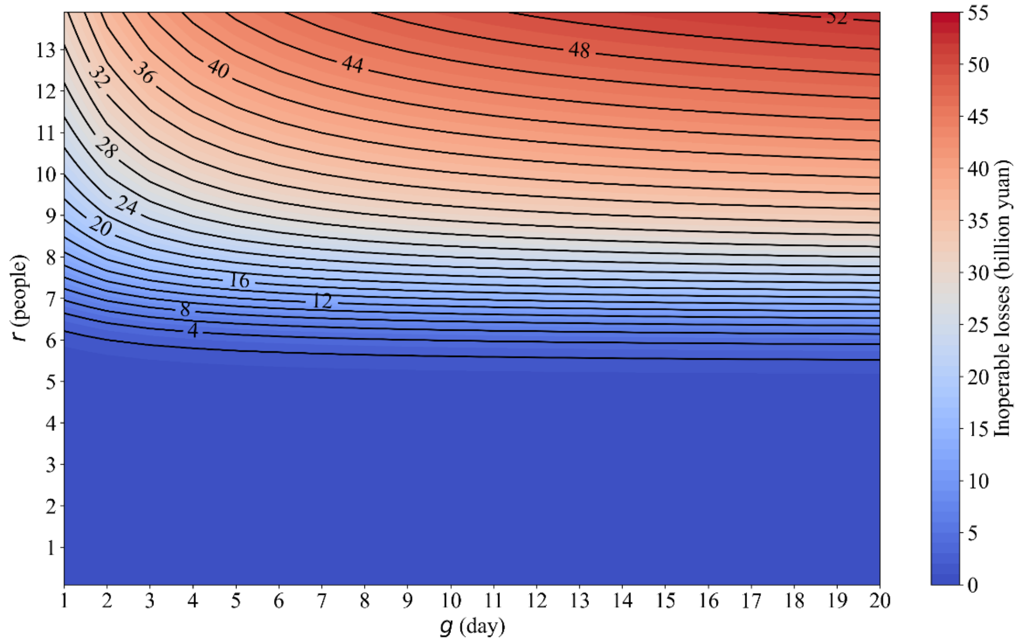

Figure S5. Inoperable losses under various control measures, where r is the effective number of daily contacts; g is the average waiting time for quarantined patients.
